# Occupation is a significant independent predictor of hypocalcemia among diabetic patients in Jinja, Uganda

**DOI:** 10.1371/journal.pone.0347799

**Published:** 2026-08-28

**Authors:** Abdirahman Ibrahim Said, Mustafe Hussein, Stephen Charles Olet, Abshir Mohamoud Hersi, Amatarahman Ibrahim Said, Said Ibrahim Said

**Affiliations:** 1 College of Health Sciences, School of Medicine, Amoud University, Borama, Somaliland; 2 Department of Internal Medicine, Kampala International University, Western Campus, Ishaka, Uganda; 3 College of Health Sciences, School of Public Health, Makerere University, Kampala, Uganda; University of Vermont, UNITED STATES OF AMERICA

## Abstract

**Background:**

Electrolyte disturbances are common among patients with diabetes mellitus and contribute significantly to morbidity. Hypocalcemia, an often-overlooked abnormality, may be influenced by both medical and lifestyle factors, particularly in resource-limited settings. This study aimed to determine the prevalence of hypocalcemia and identify its associated determinants among diabetic patients attending Jinja Regional Referral Hospital in Uganda.

**Methods:**

A cross-sectional analytical study was conducted among 374 adult diabetic patients at the medical outpatient clinic of Jinja Regional Referral Hospital. Participants were consecutively enrolled, and data were collected using structured questionnaires and laboratory investigations. Serum calcium levels were measured, with hypocalcemia defined as <2.2 mmol/L. Descriptive statistics were used to determine prevalence, while bivariate and multivariate analyses (modified Poisson regression) identified factors associated with hypocalcemia. Adjusted prevalence ratios (aPR) with 95% confidence intervals (CI) were reported, with statistical significance set at *p* < 0.05.

**Results:**

The prevalence of hypocalcemia was 28.9% (95% CI: 24.5–33.7). At bivariate analysis, significant associations were observed with occupation, social support, duration of diabetes, BMI, waist circumference, random blood glucose, and medication adherence. However, in multivariate analysis, only occupation remained independently associated with hypocalcemia. Civil servants had a significantly higher prevalence compared to farmers (aPR = 1.91; 95% CI: 1.07–3.43; *p* = 0.030).

**Conclusion:**

Hypocalcemia is highly prevalent among diabetic patients in this setting. Occupation, particularly indoor-based work such as civil service, is an independent predictor, likely reflecting reduced sunlight exposure and possible vitamin D deficiency. Routine screening for calcium abnormalities and targeted interventions addressing lifestyle factors are recommended to improve comprehensive diabetes care.

## Introduction

Diabetes mellitus (DM) continues to be the most prevalent metabolic disorder worldwide, presenting a growing public health challenge [1]. The prevalence has been rising steadily for decades, particularly in low- and middle-income countries [2]. Globally, the number of adults diagnosed with DM nearly quadrupled from 108 million in 1980 to approximately 422 million by 2014, with a corresponding increase in adult prevalence from 4.7% to 8.5% [3,4].

This increasing incidence ensures that diabetes remains a leading cause of morbidity and mortality for the foreseeable future [5]. The severity of diabetes lies in its associated complications, which can be life-threatening and irreversible if not identified and managed promptly [6]. Electrolyte imbalances represent a common and critical complication in patients with DM due to multifactorial pathophysiological factors related to the disease itself, its complications, and certain medications [3]. Electrolytes play a vital role in cellular function, enzymatic reactions, and maintaining overall homeostasis [7]. Studies across different regions highlight the widespread prevalence of these abnormalities; for instance, a study in Bangladesh found electrolyte disturbances in 78% of hospitalized DM patients [8]. Similarly, research in Africa has indicated that more than 40% of DM patients suffer from various electrolyte abnormalities [9].

Among electrolyte imbalances, hypocalcemia (low serum calcium) is a common biochemical abnormality that can present with varying degrees of severity, from asymptomatic mild cases to acute, life-threatening crises [10]. The body employs a complex homeostatic system to maintain serum calcium levels within a narrow physiological range (2.1 to 2.6 mmol/L) [10]. This regulation is primarily orchestrated by three key hormones: parathyroid hormone (PTH), vitamin D, and calcitonin, which exert specific effects on the bowel (for absorption), kidneys (for reabsorption/excretion), and skeleton (for bone remodeling) [10,11]. It is important to note that only a portion of total serum calcium is physiologically active (ionized calcium), as approximately half of total calcium is bound to protein. [10,11].

The manifestations of hypocalcemia vary significantly depending on the rate and severity of the calcium reduction, ranging from mild paresthesia, muscle spasms, cramps, and circumoral numbness to severe symptoms like tetany and seizures [10–12]. In acute cases, patients may experience laryngospasm, neuromuscular irritability, cognitive impairment, or potentially life-threatening cardiac complications such as prolonged QT intervals and electrocardiographic changes mimicking myocardial infarction or heart failure [11,12]. Chronic hypocalcemia is most commonly caused by vitamin D inadequacy, hypoparathyroidism, or resistance to these hormones [10,13]. While conventional therapy with calcium and vitamin D supplements aims to alleviate symptoms, it is associated with long-term complications such as hypercalciuria, nephrocalcinosis, renal impairment, and soft tissue calcification [11,12,14,15]

Hypocalcemia in diabetes mellitus is primarily associated with complications of poor glycemic control and related systemic disturbances. Acute kidney injury, often precipitated by volume depletion or sepsis, impairs renal phosphate excretion, leading to hyperphosphatemia and subsequent reduction in serum calcium levels. Similarly, chronic kidney disease—a common long-term complication of diabetes—contributes to hypocalcemia through phosphate retention and decreased vitamin D activation. Diabetic nephropathy may also present with nephrotic syndrome, where urinary loss of 25-hydroxyvitamin D and its binding protein further reduces calcium levels [16].

In addition, a relative hypoparathyroid state has been described in diabetic patients, characterized by a downward shift in the set-point for parathyroid hormone secretion. Hypomagnesemia, frequently observed in diabetes due to osmotic diuresis and inadequate intake, further exacerbates hypocalcemia by impairing parathyroid hormone secretion and inducing resistance to its action at both skeletal and renal levels [16]

Despite the known prevalence of the electrolyte imbalance in general and hypocalcemia in particularly [6], there remains a notable research gap in specific local contexts regarding outpatient populations in Uganda. At Jinja Regional Referral Hospital, medical outpatient records from the previous year indicated that while a significant number of diabetes patients were seen, electrolyte testing was infrequently performed (JRRH records 2021; unpublished). In the few patients who underwent testing, a high proportion (4 out of 5) presented with electrolyte disturbances, suggesting a potentially large number of undiagnosed cases within the wider patient population (JRRH records 2021; unpublished). The current proposed study aims to address this research gap by establishing the magnitude of hypocalcemia and identifying its associated risk factors in DM patients at Jinja Regional Referral Hospital, thereby facilitating improved surveillance and management strategies.

## Methodology

### Study design

This study employed a cross-sectional descriptive and analytical facility-based design. The research aimed to determine the prevalence of hypocalcemia and identify associated medical and demographic risk factors among diabetic patients at a specific point in time.

### Study setting

The study was conducted at the medical outpatient clinic of Jinja Regional Referral Hospital (JRRH) in Eastern Uganda. JRRH, founded in the 1930s, serves as one of Uganda’s thirteen Regional Referral Hospitals and provides specialist medical and surgical services, including an internal medicine department and a dedicated diabetic clinic that attends to approximately 80–100 patients per week. The hospital’s laboratory facilities were deemed adequate for conducting the necessary biochemical assessments.

### Study population

The target population for this study comprised all diabetes mellitus patients, both known and newly diagnosed, attending the medical outpatient clinic at Jinja Regional Referral Hospital. The accessible population included all DM patients aged 18 years and above who presented to the clinic during the study period.

### Eligibility criteria

- **Inclusion Criteria:** All patients with diabetes mellitus attending the medical outpatient clinic at JRRH who provided written informed consent to participate in the study.
- **Exclusion Criteria:** Patients who were acutely ill requiring emergency intervention or hospitalization during the data collection period. Additionally, individuals taking medications known to directly interfere with fluid or electrolyte homeostasis (or with pre-existing conditions known to significantly alter serum calcium levels (diabetic insipidus, severe renal failure (CKD stage 4–5), and severe heart failure) were excluded.

### Sample size calculation

The sample size was determined using the Daniel formula for calculating sample size for a single proportion (prevalence of overall electrolyte disturbance).

The formula used was: n = z²p(1-p)/d²

Where:

- n = Desired sample size.
- z = Z-statistic corresponding to a 95% level of confidence (1.96).
- p = Overall prevalence of electrolyte disturbance in a similar population. Based on a study conducted in Ethiopia by Woyesa et al. (2019) (12), the overall prevalence of electrolyte disturbance was 42.0%.
- d = Level of precision (set at 0.05).

**Calculation:**n = (1.96)² × 0.42 × (1–0.42) / (0.05)²n = (3.8416 × 0.42 × 0.58) / 0.0025n = 0.9351 / 0.0025n = 374

Therefore, a sample size of 374 participants was required for the study.

### Sampling procedure

Consecutive enrollment was utilized to recruit all eligible study participants attending the medical outpatient clinic at JRRH. Participants were enrolled sequentially as they presented to the clinic and met the eligibility criteria until the required sample size of 374 was achieved.

### Data collection instruments

Data was collected using a pre-tested, structured, investigator-administered questionnaire and laboratory report forms. The questionnaire included sections on socio-demographic characteristics (age, sex, residence, occupation, education level, marital status, alcohol consumption), medical factors (duration since DM diagnosis, BMI, waist circumference, waist-to-hip ratio, coexistent hypertension, coexistent HIV status, adherence to treatment, type of antidiabetic medication), and lifestyle factors. And The study a period was two months; from July 2022 to Augst 2022. The duration was be adequate to achieve the study sample size.

### Laboratory procedures and measurement

- **Biochemical analysis:** A blood sample of approximately 5 mL was drawn from each participant using an aseptic technique. The sample was taken to the JRRH chemistry laboratory for analysis of serum electrolytes, specifically serum calcium levels.
- **Serum Sodium Measurement:** The blood samples were centrifuged using a K102a centrifuge at 4200 r/minute for 2 minutes. The serum was then analyzed using a Semi-Auto chemistry analyzer (Phoenix Brand) or a chemistry machine (Cobas-C311). Hypocalcemia was defined according to international reference ranges as a serum calcium concentration less than < 2.2 mmol/L (WHO, 2021).
- **Blood Glucose Measurement:** Random blood glucose levels were measured using a glucometer (One Touch brand) following standard procedures, ensuring proper calibration and test strip validity.
- **Anthropometric Measurements:** Height and weight were measured using calibrated weighing scales and height boards (HFMED brand). Body Mass Index (BMI) was calculated (weight in kg divided by height in meters squared). Waist circumference and waist-to-hip ratio were measured using a tape measure according to international standards (WHO, 2008; CDC, 2022).

### Data analysis

Data from questionnaires were entered into Microsoft Excel version 2010 and then exported to STATA version 14.2 for analysis.

- **Descriptive Analysis:** The prevalence of hypocalcemia was calculated as a percentage of the total study population and presented using frequencies and percentages.
- **Bivariate Analysis:** Chi-square tests were employed to assess the association between socio-demographic and medical factors (independent variables) and the occurrence of hypocalcemia (dependent variable). Variables with a significance level of P < 0.20 at bivariate analysis were carried forward to multivariate analysis.
- **Multivariate Analysis:** Modified Poisson regression analysis was applied to identify factors independently associated with hypocalcemia, adjusting for potential confounders. A forward stepwise regression technique was used to select the best-fitting model. The strength of association was evaluated using adjusted prevalence ratios (aPR) with corresponding 95% confidence intervals (CI), and a P-value less than 0.05 was considered statistically significant.

### Ethical considerations

The study was approved by the Kampala International University Research Ethics Committee **(Approval No.** *KIU-2022–117***).** Written informed consent was obtained from all participants before enrollment.

## Results

This section presents the findings from the descriptive and inferential analysis of the study data collected from 374 participants attending the medical outpatient clinic at Jinja Regional Referral Hospital.

### Socio-demographic and clinical characteristics of study participants

A total of 374 participants were included in the study. The socio-demographic characteristics show that the study population was predominantly female (67.11%, n = 251). The mean age of participants was approximately 50 years, with the largest group falling into the 46–60 years age category (46.26%, n = 173).

Most participants reported having primary education (42.25%, n = 158), while 28.34% (n = 106) had no formal education. The majority of participants resided in rural areas (64.17%, n = 240). The most common occupation reported was “Others” (40.11%, n = 150), followed by “Farmer” (34.76%, n = 130). Over half of the participants reported being married (56.15%, n = 210).

Regarding income, a significant portion of participants (65.51%, n = 245) reported earning less than 200,000 Ugandan shillings monthly. The median duration since diabetes diagnosis was 1–5 years for 45.72% (n = 171) of participants, with 24.06% (n = 90) having lived with the condition for 10–20 years.

A high percentage of participants (82.62%, n = 309) reported high medication adherence, while 10.16% (n = 38) reported low adherence. The majority of participants were taking oral antidiabetic medication (57.75%, n = 216). Table 1 provides a detailed summary of the baseline characteristics.

**Table 1:** Socio-demographic and clinical characteristics of participants (n = 374).

Characteristic	Category	Frequency (n)	Percentage (%)
**Gender**	Male	123	32.89
	Female	251	67.11
**Age Category**	16-30 years	22	5.88
	31-45 years	81	21.66
	46-60 years	173	46.26
	61-75 years	87	23.26
	>75 years	11	2.94
**Education Level**	No formal education	106	28.34
	Primary	158	42.25
	Secondary and above	110	29.41
**Area of Residence**	Rural	240	64.17
	Urban	134	35.83
**Occupation**	Farmer	130	34.76
	Civil service	20	5.35
	Business man/woman	74	19.79
	Others	150	40.11
**Marital Status**	Single	104	27.81
	Married	210	56.15
	Widowed	55	14.71
	Divorced	5	1.34
**Average Monthly Income**	<200,000	245	65.51
	200,000-450,000	76	20.32
	450,000-900,000	38	10.16
	>900,000	15	4.01
**Duration Since Diagnosis**	1-5 years	171	45.72
	5-10 years	85	22.73
	10-20 years	90	24.06
	20-30 years	22	5.88
	More than 30 years	6	1.60
**BMI**	Normal	230	61.50
	>25 (Overweight/Obese)	124	33.16
	<18 (Underweight)	20	5.35
**Waist Circumference**	Yes (>40M, 35F)	130	34.76
	No	244	65.24
**Waist-to-Hip Ratio**	Yes (>94M, 80F)	139	37.17
	No	235	62.83
**Coexistent Hypertension**	Yes	267	71.39
	No	107	28.61
**Coexistent HIV Status**	Yes	49	13.10
	No	312	83.42
	Unknown	13	3.48
**Type of Antidiabetic Medication**	Insulin	123	32.89
	Oral	216	57.75
	Mixed	31	8.29
	None	4	1.07
**Medication Adherence**	Low	38	10.16
	Medium	27	7.22
	High	309	82.62
**Social Support**	Yes	223	59.63
	No	151	40.37

### Prevalence of hypocalcemia among diabetic patient

The overall proportion estimation from the study indicates that **28.9% (n = 108**) of participants presented with hypocalcemia, with a 95% confidence interval ranging from 24.5% to 33.7%. The majority of participants**, 71.1% (n = 266),** had no hypocalcemia.

### Result: Bivariate analysis of factors associated with hypocalcemia (n=374)

This section presents the findings from the bivariate analysis, identifying potential associations between hypocalcemia status and various socio-demographic, medical, and lifestyle factors.

1. Overall Prevalence of Hypocalcemia

Out of the total 374 participants, 108 (28.88%) presented with hypocalcemia (serum calcium level < 2.2 mmol/L), while 266 (71.12%) had normal or high serum calcium levels.

1. Socio-demographic Factors

Table 2 A details the prevalence of hypocalcemia across different socio-demographic characteristics of the study participants.

**Table 2:** A. Bivariate analysis of socio-demographic factors and hypocalcemia.

Characteristic	Category	No Hypocalcemia (n = 266) %	Hypocalcemia (n = 108) %	P-value (Chi-square)
**Gender**	Male (1)	87 (70.73)	36 (29.27)	0.887
	Female (2)	179 (71.31)	72 (28.69)	
**Age Category**	16-30 years (1)	16 (72.73)	6 (27.27)	0.771
	31-45 years (2)	55 (67.90)	26 (32.10)	
	46-60 years (3)	128 (73.99)	45 (26.01)	
	61-75 years (4)	59 (67.82)	28 (32.18)	
	>75 years (5)	8 (72.73)	3 (27.27)	
**Education Level**	No formal education (1)	77 (72.64)	29 (27.36)	0.814
	Primary (2)	110 (69.62)	48 (30.38)	
	Secondary and above (3)	79 (71.82)	31 (28.18)	
**Area of Residence**	Rural (1)	178 (74.17)	62 (25.83)	**0.052** *
	Urban (2)	88 (65.67)	46 (34.33)	
**Occupation**	Farmer (1)	99 (76.15)	31 (23.85)	**0.021** *
	Civil service (2)	9 (45.00)	11 (55.00)	
	Business man/woman (3)	51 (68.92)	23 (31.08)	
	Others (4)	107 (71.33)	43 (28.67)	
**Average Monthly Income**	<200,000 (1)	178 (72.65)	67 (27.35)	0.143
	200,000-450,000 (2)	51 (67.11)	25 (32.89)	
	450,000-900,000 (3)	24 (63.16)	14 (36.84)	
	>900,000 (4)	13 (86.67)	2 (13.33)	
**Marital Status**	Single (1)	71 (68.27)	33 (31.73)	0.380
	Married (2)	149 (70.95)	61 (29.05)	
	Widowed (3)	41 (74.55)	14 (25.45)	
	Divorced (4)	5 (100.00)	0 (0.00)	
**Social Support**	Yes (1)	168 (75.34)	55 (24.66)	**0.019** *
	No (2)	98 (64.90)	53 (35.10)	
**Alcohol Frequency**	Never (1)	232 (70.95)	95 (29.05)	0.963
	Monthly or less (2)	15 (75.00)	5 (25.00)	
	2-4 times a month (3)	7 (70.00)	3 (30.00)	
	2-3 times a week (4)	9 (69.23)	4 (30.77)	
	4 or more times a week (5)	3 (75.00)	1 (25.00)	

*Note: P-values have been calculated based on the data provided for this analysis.

### Summary of Findings (Socio-demographic Factors)

The bivariate analysis identified statistically significant associations between hypocalcemia and area of residence (P = 0.052), occupation (P = 0.021), and social support for diabetes care (P = 0.019). No significant association was observed for gender, age category, level of education, marital status, or alcohol consumption frequency.

- **Residence:** The prevalence of hypocalcemia was notably higher among urban residents (34.33%) compared to rural residents (25.83%).
- **Occupation:** Participants employed in civil service exhibited the highest prevalence of hypocalcemia (55.00%), more than double that of farmers (23.85%) and significantly higher than business owners/women (31.08%) or those in the “Others” category (28.67%).
- **Social Support:** Participants who reported having no social support for their diabetes care had a significantly higher prevalence of hypocalcemia (35.10%) compared to those who did have support (24.66%).
- Income: While not statistically significant (P = 0.143), the prevalence generally increased with income, reaching a peak in the 450,000–900,000 UGX category (36.84%), but then dropped sharply in the highest income group (>900,000 UGX) (13.33%).

### Medical, clinical, and lifestyle factors

Table 3 **B** presents the associations between hypocalcemia and various medical conditions, lifestyle habits, and anthropometric measures.

**Table 3:** B. Bivariate analysis of medical, clinical, and lifestyle factors and hypocalcemia.

Characteristic	Category	No Hypocalcemia (n = 266) %	Hypocalcemia (n = 108) %	P-value (Chi-square)
**Duration Since Diagnosis**	1-5 years (1)	123 (71.93)	48 (28.07)	**0.003** *
	5-10 years (2)	66 (77.65)	19 (22.35)	
	10-20 years (3)	60 (66.67)	30 (33.33)	
	20-30 years (4)	14 (63.64)	8 (36.36)	
	>30 years (5)	3 (50.00)	3 (50.00)	
**BMI**	Normal (1)	154 (66.96)	76 (33.04)	**0.021** *
	>25 (Overweight/Obese) (2)	95 (76.61)	29 (23.39)	
	<18 (Underweight) (3)	17 (85.00)	3 (15.00)	
**Waist Circumference**	Yes (>40M, 35F) (1)	104 (80.00)	26 (20.00)	**0.003** *
	No (2)	162 (66.39)	82 (33.61)	
**Coexistent Hypertension**	Yes (1)	185 (69.29)	82 (30.71)	0.170
	No (2)	81 (75.70)	26 (24.30)	
**Random Blood Sugar**	<10 mmol/l (1)	138 (67.32)	67 (32.68)	**0.040** *
	>10 mmol/l (2)	128 (75.74)	41 (24.26)	
**Type of Antidiabetic Medication**	Insulin (1)	88 (71.54)	35 (28.46)	0.499
	Oral (2)	156 (72.22)	60 (27.78)	
	Mixed (3)	20 (64.52)	11 (35.48)	
	None (4)	2 (50.00)	2 (50.00)	
**Adherence (Categorical)**	Low (1)	28 (73.68)	10 (26.32)	0.725
	Medium (2)	21 (77.78)	6 (22.22)	
	High (3)	217 (70.23)	92 (29.77)	
**Adherence (Adherencetot)**	Low (Cat 4)	8 (61.54)	5 (38.46)	**0.048** *
	High (Cat 7)	207 (71.13)	84 (28.87)	

*Note: P-values have been calculated based on the data provided for this analysis.

### Summary of findings (Medical, clinical, and lifestyle factors)

The bivariate analysis identified statistically significant associations between hypocalcemia and duration of diabetes (P = 0.003), BMI (P = 0.021), waist circumference (P = 0.003), random blood sugar levels (P = 0.040), and medication adherence (P = 0.048 for low adherence).

- **Duration of Diabetes:** A strong positive dose-response relationship was observed, with hypocalcemia prevalence increasing significantly with the duration of diabetes. The prevalence increased from 28.07% in patients diagnosed for 1–5 years to 50.00% in those diagnosed for more than 30 years.
- BMI: Hypocalcemia prevalence was highest in patients categorized as having normal BMI (33.04%), compared to overweight/obese (23.39%) and underweight (15.00%) groups. This finding suggests a more complex relationship between BMI and hypocalcemia in this specific population than often assumed.
- **Waist Circumference:** Participants without high waist circumference measurements had a higher prevalence of hypocalcemia (33.61%) compared to those with high measurements (20.00%). This finding warrants further investigation, as high waist circumference is generally associated with poor metabolic health.
- **Random Blood Sugar:** Counterintuitively, patients with controlled random blood sugar (<10 mmol/L) had a higher prevalence of hypocalcemia (32.68%) than those with uncontrolled blood sugar (>10 mmol/L) (24.26%).
- Medication Adherence: Participants reporting low adherence to medication (Cat 4 in adherencetot) had a higher prevalence of hypocalcemia (38.46%) compared to those with high adherence (28.87%). This highlights the potential role of poor self-management in electrolyte disturbances.

### Results: Multivariate analysis of factors associated with hypocalcemia

This section presents the results of the multivariate modified Poisson regression analysis. Variables that showed an association at a significance level of P < 0.20 in the bivariate analysis were included in the model to identify independent predictors of hypocalcemia. The results are presented as adjusted prevalence ratios (aPR) with 95% confidence intervals (CI).

### Multivariate modified poisson regression results for hypocalcemia (n=374)

**Table:** 

Characteristic	Categories	Prevalence Ratio (aPR)	95% Confidence Interval	P-value
**Area of Residence**	Ref: Rural	1.00		
	Urban	1.22	[0.87, 1.70]	0.247
**Occupation**	Ref: Farmer	1.00		
	Civil service	**1.91**	**[1.07, 3.43]**	0.030*
	Business man/woman	1.34	[0.80, 2.25]	0.272
	Others	1.19	[0.79, 1.80]	0.406
**Average Monthly Income**	Ref: < 200,000 UGX	1.00		
	200,000–450,000 UGX	1.09	[0.74, 1.62]	0.658
	450,000–900,000 UGX	0.96	[0.59, 1.54]	0.858
	>900,000 UGX	0.34	[0.08, 1.39]	0.134
**Duration Since Diagnosis**	Ref: 1–5 years	1.00		
	5-10 years	0.84	[0.53, 1.29]	0.464
	10-20 years	1.28	[0.87, 1.87]	0.206
	20-30 years	1.51	[0.87, 2.60]	0.142
	>30 years	1.51	[0.64, 3.54]	0.345
**BMI**	Ref: Normal	1.00		
	Overweight/Obese (>25)	0.79	[0.53, 1.17]	0.240
	Underweight (<18)	0.48	[0.17, 1.38]	0.174
**Waist Circumference**	Ref: Yes (>40M, 35F)	1.00		
	No (<40M, 35F)	1.39	[0.95, 2.04]	0.088
**Coexistent Hypertension**	Ref: Yes	1.00		
	No	0.80	[0.56, 1.21]	0.231
**Random Blood Sugar**	Ref: < 10 mmol/L	1.00		
	>10 mmol/L	0.80	[0.56, 1.13]	0.204
**Social Support**	Ref: Yes	1.00		
	No	1.28	[0.89, 1.80]	0.142
**Medication Adherence**	Ref: Low (Cat 4)	1.00		
	Cat 5	0.49	[0.16, 1.53]	0.218
	Cat 6	0.46	[0.15, 1.40]	0.163
	Cat 7	0.63	[0.29, 1.36]	0.276
	Cat 8	1.05	[0.39, 2.80]	0.927

**aPR (Adjusted Prevalence Ratio) calculated by exponentiating the coefficient (exp(coef.)) from the GLM model; Reference categories are based on STATA’s default (lowest numbered category).

### Summary of multivariate findings

After adjusting for other variables in the multivariate model, only **occupation** showed a statistically significant association with hypocalcemia.

- Occupation: Participants employed in civil service (Category 2) had a **1.91 times higher prevalence of hypocalcemia** compared to farmers (reference category, Category 1) (aPR = 1.91, 95% CI: 1.07–3.43, P = 0.030).
- Non-significant Factors: Several variables that appeared to have an association in the bivariate analysis (e.g., area of residence, social support, waist circumference, and random blood sugar levels) were no longer statistically significant in the multivariate model. This suggests that their initial association was likely confounded by other factors in the model.
- BMI: While not statistically significant, the prevalence of hypocalcemia among underweight participants (<18 BMI) was nearly half that of normal BMI participants (aPR = 0.48, 95% CI: 0.17–1.38, P = 0.174).

## Discussion

The present study investigated the determinants of hypocalcemia in a population of diabetic patients at Jinja Regional Referral Hospital in Uganda. Our findings indicate a notable prevalence of hypocalcemia, detected in 28.9% (n = 108) of participants, highlighting a common and critical electrolyte disturbance within this patient group. The multivariate analysis identified occupation as a significant independent predictor of hypocalcemia.

Specifically, participants employed in civil service exhibited a 1.91 times higher prevalence of hypocalcemia compared to farmers (aPR = 1.91, 95% CI: 1.07–3.43, P = 0.030). This finding points to a complex interplay between medical conditions and specific lifestyle or environmental factors that influence calcium homeostasis, particularly through the lens of vitamin D status.

The link between occupation and hypocalcemia in diabetic patients is often mediated by vitamin D deficiency. Vitamin D plays an essential role in regulating calcium homeostasis [17]. As detailed in the literature, vitamin D is crucial for intestinal calcium absorption. The active form of vitamin D, calcitriol, interacts with vitamin D receptors (VDRs) in enterocytes to promote the absorption of dietary calcium into the bloodstream [17]. Therefore, inadequate vitamin D status directly impairs the body’s ability to maintain optimal serum calcium levels through dietary intake alone.

The impact of occupation on vitamin D levels, and subsequently on calcium homeostasis, has been extensively researched in various populations. Studies demonstrate that individuals who spend extended periods indoors, such as indoor workers and shiftworkers, experience significantly lower serum vitamin D levels compared to outdoor workers [18]. This difference is primarily attributed to decreased exposure to solar ultraviolet B (UVB) radiation, which is essential for endogenous vitamin D synthesis in the skin [17,18]. The Sowah et al. review highlights that indoor occupations are associated with high risks of vitamin D deficiency and insufficiency [18].

Our finding that civil service employees face a higher risk of hypocalcemia (aPR = 1.91) aligns with this pattern, as these roles typically restrict daytime sunlight exposure. The resulting vitamin D deficiency in this group likely reduces calcium absorption efficiency, contributing to hypocalcemia [17].

While our bivariate analysis identified several significant associations with hypocalcemia (e.g., area of residence, social support, and duration of diabetes), the multivariate model suggests that occupation remains a key independent factor. For instance, the inverse relationship found between hypocalcemia and waist circumference/BMI in our bivariate results (Table 3), while counterintuitive based on general metabolic assumptions, warrants further investigation to understand potential confounding factors or unique aspects of this specific population.

The clinical significance of hypocalcemia in diabetic patients extends beyond simple mineral imbalance. As noted by Babikr et al. (2016), hypocalcemia and hypomagnesemia can serve as prognostic indicators for poor glycemic control in type 2 diabetes [19]. The current study also supports a complex metabolic relationship, evidenced by significant bivariate correlations between serum calcium levels and medical factors such as HbA1c, duration of diabetes, and random blood sugar (Tables 2 and 3).

The intricate connection between calcium and metabolic dysfunction is further supported by Li et al. (2015), who found that serum calcium levels positively correlated with various metabolic parameters including fasting plasma glucose, HOMA-IR, and dyslipidemia in diabetic patients [18]. This relationship is particularly concerning given that hypocalcemia is linked to adverse outcomes in patients with comorbidities like diabetes and COVID-19 [20]

### Study limitations

This study has several limitations. Because of its cross-sectional design, causal relationships cannot be established. Serum vitamin D and parathyroid hormone concentrations were not measured, limiting mechanistic interpretation. The study was conducted at a single referral hospital, which may limit generalizability.

## Conclusion

Our study at Jinja Regional Referral Hospital identifies a high prevalence of hypocalcemia among diabetic patients and establishes occupation, specifically civil service, as a significant determinant. Given the critical role of vitamin D in regulating calcium homeostasis and the link between occupational factors and vitamin D levels, screening for and addressing hypocalcemia and potential vitamin D deficiency in diabetic patients is essential for comprehensive management. These findings highlight a need for researchers and clinicians to recognize these specific risks in vulnerable patient groups and consider targeted interventions, especially for those in indoor occupations.

## Supporting information

S1 Dataset Minimal calcium dataset. This file contains the clinical, demographic, and biochemical data of the study participants used to analyze the prevalence and predictors of hypocalcemia.
(XLSX)
