# Supplementary material for: Occupation is a significant independent predictor of hypocalcemia among diabetic patients in Jinja, Uganda
Source: PLoS One. 2026 Aug 28;21(8):e0347799. doi: 10.1371/journal.pone.0347799 (PMC13524234; doi:10.1371/journal.pone.0347799)
Supplement: Supplementary file 1 [file pone.0347799.s002.docx]

Date: 4/8/2026

**To:** Gary S. Stein, Academic Editor, PLOS ONE

**From:** Dr. Abdirahman Ibrahim Said

**Subject:** Response to Reviewers for PONE-D-26-16270

**Title:** Occupation is aSignificant Independent Predictor of Hypocalcemia Among Diabetic Patients in Jinja, Uganda

Dear Dr. Stein,

Thank you for the opportunity to revise our manuscript. We appreciate the constructive feedback provided by the editorial team and the reviewer. We have addressed all requirements and comments below.

**PART 1: Response to Journal Requirements**

1. Style and Formatting:

- Requirement: Ensure the manuscript meets PLOS ONE’s style requirements and

file naming.

- Response: We have reformatted the manuscript to strictly follow the PLOS ONE

template.

2. Data Availability:

- Requirement: Provide a non-author contact or make data fully available.

- Response: We have decided to make the data fully available to ensure

complete transparency and compliance with PLOS ONE policy. We have uploaded

the minimal data set as a Supporting Information file labeled **" data calcium ".**

3. Reference List:

- Requirement: Review the reference list for completeness and correctness.

- Response: We have thoroughly checked the reference list. All citations match

the text, and we have confirmed that no retracted papers are cited.

**PART 2: Response to Reviewer #1 (James Natweta Baguma**)

Comment 1: "I realize you have missed out in line 142 indicating the reference number of the Makerere University School of Social Sciences Research and Ethics Committee. Please indicate it given it leads you to the UNCST approval." Response: Thank you for this important observation. We would like to clarify that while one of our co-authors (Stephen Charles Olet) is affiliated with Makerere University, the ethical approval for this study was granted by the Kampala International University Research Ethics Committee (KIU-REC). In response to your request, we have added the official protocol number to the "Ethical Considerations" section It now reads: "Institutional approval was obtained from the Kampala International University Research Ethics Committee (KIU-REC) with Protocol number **KIU-2022-117,** and administrative clearance was granted by Jinja Regional Referral Hospital." We believe these revisions have significantly strengthened the manuscript and addressed all concerns. We look forward to your positive decision regardingpublication in PLOS ONE.

**Sincerely,**

Dr. Abdirahman Ibrahim Said

Corresponding Author Amoud University, Somalia
